# Supplementary material for: Mode of action of the antimicrobial peptide Mel4 is independent of Staphylococcus aureus cell membrane permeability
Source: PLoS One. 2019 Jul 29;14(7):e0215703. doi: 10.1371/journal.pone.0215703 (PMC6663011; doi:10.1371/journal.pone.0215703)
Supplement: S1 Table — Cell membrane depolarization as assessed by the release of the membrane potential sensitive dye DiSC3-5, measured spectroscopically at 622nm excitation and 670nm emission wavelengths. Data are presented as means (±SD) of three independent repeats performed in triplicate. (PDF) [file pone.0215703.s001.pdf]

S1 Table. Cell membrane depolarization. Cell membrane depolarization as assessed by the release of the membrane potential sensitive dye DiSC3-5, measured spectroscopically at 622_{nm} excitation and 670_{nm} emission wavelengths. Data are presented as means (±SD) of three independent repeats performed in triplicate.

Time (sec)	*S. aureus* 31						*S. aureus* ATCC 6538					
	Melimine		Mel4		DMSO 20%	Buffer	Melimine		Mel4		DMSO 20%	Buffer
	1X	2X	1X	2X			1X	2X	1X	2X		
**30**	8±1	11±2	13±2	13±2	21±2	1±0	7±3	11±6	11±2	11±3	21±2	1±0
**60**	15±3	18±2	17±4	18±2	26±2	2±1	12±3	15±2	15±2	16±1	26±2	2±1
**90**	18±1	21±2	21±3	23±4	31±2	3±1	17±3	19±3	19±2	21±2	31±2	3±1
**120**	23±4	27±2	27±4	27±7	39±2	3±1	22±4	25±3	26±2	29±2	39±2	3±1
**150**	25±1	29±2	35±5	32±3	45±4	3±1	24±3	27±4	29±2	32±3	45±4	3±1
**180**	28±2	32±4	36±5	38±1	49±5	3±1	27±3	30±4	33±1	37±3	49±5	3±1
**210**	32±3	34±4	38±2	41±1	52±7	3±1	30±3	33±2	35±1	40±1	52±7	3±1
**240**	35±3	38±2	41±1	46±3	65±4	3±1	33±2	34±1	39±1	46±2	65±4	3±1
**270**	40±2	42±4	47±4	49±2	67±2	3±1	37±3	41±4	42±2	48±1	67±2	3±1
**300**	44±2	48±3	57±4	53±4	68±2	3±1	42±3	47±4	47±3	49±1	68±2	3±1
