# Supplementary material for: Mode of action of the antimicrobial peptide Mel4 is independent of Staphylococcus aureus cell membrane permeability
Source: PLoS One. 2019 Jul 29;14(7):e0215703. doi: 10.1371/journal.pone.0215703 (PMC6663011; doi:10.1371/journal.pone.0215703)
Supplement: S2 Table — The decrease in the number of viable bacteria (CFU/ml) that occurred at the same time as measuring changes to their membrane depolarization. Data are presented as means (±SD) of three independent repeats performed in triplicate. (PDF) [file pone.0215703.s002.pdf]

S2 Table. Reduction in number of live bacteria during treatment with the peptides. The decrease in the number of viable bacteria (CFU/ml) that occurred at the same time as measuring changes to their membrane depolarization. Data are presented as means (±SD) of three independent repeats performed in triplicate.

Time (sec)	*S. aureus* 31 ATCC 6538					*S. aureus* ATCC 6538				
	Melimine		Mel4		Buffer	Melimine		Mel4		Buffer
	1X	2X	1X	2X		1X	2X	1X	2X	
**30**	7080450/636	6903000/4243	7003500/4950	6900500/707	10000000/1789	7080500/707	6901500/2121	7002000/2828	6902000/2828	10000000/3342
**60**	6903000/4243	6603500/4950	6867666/1414	6756555/1414	10000000/2908	6902000/2828	660200/2828	6867166/707	6757555/2828	10000000/5324
**90**	6520000/495	6303500/4950	6768166/2121	6754555/1414	10000000/6788	6502000/2828	630350/4950	6767666/1414	6756055/707	10000000/4457
**120**	6104500/6364	5901500/2121	6465666/1414	6353555/2828	10000000/4473	6103000/4243	5904000/5657	6468166/2121	6357555/2828	10000000/7845
**150**	5904500/6364	5503500/4950	6051666/21213	5956555/1414	10000000/1373	5902500/3536	5503500/4950	6068166/2121	5957555/2828	10000000/4003
**180**	5786333/4243	5125722/4950	5846666/28284	5857555/2828	10000000/9832	5784833/2121	5123722/2121	5866166/707	5856055/707	10000000/6992
**210**	5202500/3536	4803000/4243	5664166/3536	5557055/2121	10000000/1841	5203500/4950	4802000/2828	5667666/1414	5555755/283	10000000/9802
**240**	5086333/4243	4324722/3536	5465166/2121	5456055/707	10000000/9098	5083383/71	4325722/4950	5468166/2121	5454555/1414	10000000/7982
**270**	4984333/1414	4125222/4243	5064166/3536	5054055/2121	10000000/1411	4983333/0	4124222/2828	5067166/707	5057555/2828	10000000/2552
**300**	4684833/2121	3825222/4243	4864166/3536	5155055/707	10000000/3454	4685333/2828	3824722/3536	4865666/1414	4757055/2121	10000000/2526
