# Supplementary material for: Mode of action of the antimicrobial peptide Mel4 is independent of Staphylococcus aureus cell membrane permeability
Source: PLoS One. 2019 Jul 29;14(7):e0215703. doi: 10.1371/journal.pone.0215703 (PMC6663011; doi:10.1371/journal.pone.0215703)
Supplement: S3 Table — Cell membrane permeabilization was assessed as the increase in fluorescence intensity due to interaction of Sytox green dye with DNA (measured spectroscopically at 480nm excitation and 522nm emission wavelengths). Data are presented as means (±SD) of three independent repeats performed in triplicate. (PDF) [file pone.0215703.s003.pdf]

S3 Table. Cell membrane permeabilization caused by the two peptides. Cell membrane permeabilization was assessed as the increase in fluorescence intensity due to interaction of Sytox green dye with DNA (measured spectroscopically at 480_{nm} excitation and 522_{nm} emission wavelengths). Data are presented as means (±SD) of three independent repeats performed in triplicate.

Time (sec)	*S. aureus* 31						*S. aureus* ATCC 6538					
	Melimine		Mel4		Triton-X (1%)	Buffer	Melimine		Mel4		Triton- X (1%)	Buffer
	1X	2X	1X	2X			1X	2X	1X	2X		
**5**	4±1	2±0	-2±1	-2±2	33±1	1±2	2±1	2±1	1±0	1±0	17±1	1±1
**10**	3±1	5±1	-3±3	-3±2	42±2	1±2	4±1	5±0	2±0	2±0	38±1	2±1
**15**	6±1	6±0	-3±1	-3±3	57±3	1±2	9±1	10±1	2±1	2±0	48±1	1±2
**20**	7±2	6±1	-4±1	-4±2	67±1	1±1	13±2	16±2	2±1	2±1	60±3	1±1
**25**	11±1	11±1	-3±2	-4±1	88±1	1±2	15±1	16±0	2±0	1±1	70±2	1±2
**30**	14±2	14±2	-5±1	-5±2	104±1	1±1	22±1	22±1	3±1	4±2	80±2	2±1
**150**	33±4	34±2	-2±1	-1±1	147±7	1±2	41±2	44±2	8±1	10±	155±14	2±2
