# Supplementary material for: Mode of action of the antimicrobial peptide Mel4 is independent of Staphylococcus aureus cell membrane permeability
Source: PLoS One. 2019 Jul 29;14(7):e0215703. doi: 10.1371/journal.pone.0215703 (PMC6663011; doi:10.1371/journal.pone.0215703)
Supplement: S4 Table — The leakage of cellular ATP (in percentage) from bacteria after treatment with 1X and 2X MIC of each peptide. Data are presented as means (±SD) of three independent repeats performed in triplicate. (PDF) [file pone.0215703.s004.pdf]

S4 Table. Leakage of ATP from cells upon addition of the two peptides. The leakage of cellular ATP (in percentage) from bacteria after treatment with 1X and 2X MIC of each peptide. Data are presented as means (±SD) of three independent repeats performed in triplicate.

Time (min)	*S. aureus* 31					*S. aureus* ATCC 6538				
	Melimine		Mel4		Buffer	Melimine		Mel4		Buffer
	1X	2X	1X	2X		1X	2X	1X	2X	
**0**	0±0	0±0	0±0	0±0	0±0	0±0	0±0	0±0	0±0	0±0
**2**	53±2	57±1	21±1	21±1	2±0	38±3	39±2	16±2	17±2	2±0
**4**	56±0	59±1	21±2	22±1	2±1	38±3	40±2	17±2	18±1	2±1
**6**	58±1	60±1	23±0	23±2	2±1	40±3	41±2	18±1	19±1	2±1
**8**	58±1	60±1	23±1	23±2	2±1	40±3	42±2	19±2	20±1	2±1
**10**	60±2	61±2	23±1	23±2	2±1	40±3	42±2	19±2	20±1	2±1
