# Supplementary material for: Mode of action of the antimicrobial peptide Mel4 is independent of Staphylococcus aureus cell membrane permeability
Source: PLoS One. 2019 Jul 29;14(7):e0215703. doi: 10.1371/journal.pone.0215703 (PMC6663011; doi:10.1371/journal.pone.0215703)
Supplement: S5 Table — Data are presented as means (±SD) of three independent repeats performed in triplicate. (PDF) [file pone.0215703.s005.pdf]

S5 Table. **Reduction in number of live bacteria during treatment with peptides that occurred in the ATP assay.** Data are presented as means (±SD) of three independent repeats performed in triplicate.

Time (min)	*S. aureus* 31					*S. aureus* ATCC 6538				
	Melimine		Mel4		Buffer	Melimine		Mel4		Buffer
	1X	2X	1X	2X		1X	2X	1X	2X	
**2**	310113/196	292963/3496	2267747/29251	2247990/35610	10000000/567	364853/9781	324444/25513	3690778/286927	3373333/219235	10000000/654
**4**	303133/12241	286000/4582	2229347/10835	2258647/39599	10000000/246	331573/6096	323829/8978	3244459/1937	3244368/1332	10000000/987
**6**	286067/569	286790/1018	2225161/10257	2234490/29892	10000000/873	328013/5955	317724/5715	3223781/19032	3231852/17650	10000000/569
**8**	276640/13650	284848/2451	2241837/25830	2234467/18927	10000000/141	330770/12903	323882/2673	3242592/32960	3138481/12158	10000000/252
**10**	284797/2691	283017/4260	2229450/15439	2212663/1363	10000000/754	325939/2438	318219/10185	3123348/1145	3122481/1198	10000000/179
