# Supplementary material for: Mode of action of the antimicrobial peptide Mel4 is independent of Staphylococcus aureus cell membrane permeability
Source: PLoS One. 2019 Jul 29;14(7):e0215703. doi: 10.1371/journal.pone.0215703 (PMC6663011; doi:10.1371/journal.pone.0215703)
Supplement: S6 Table — The release of UV absorbing materials (DNA/RNA) was determined spectroscopically at OD260nm after treatment with 1X and 2X MIC of peptides. Data are presented as means (±SD) of three independent repeats performed in triplicate. (PDF) [file pone.0215703.s006.pdf]

S6 Table. Release of DNA/RNA from cells upon addition of peptides. The release of UV absorbing materials (DNA/RNA) was determined spectroscopically at OD_{260nm} after treatment with 1X and 2X MIC of peptides. Data are presented as means (±SD) of three independent repeats performed in triplicate.

Time (min)	*S. aureus* 31					*S. aureus* ATCC 6538				
	Melimine		Mel4		Buffer	Melimine		Mel4		Buffer
	1X	2X	1X	2X		1X	2X	1X	2X	
0	1.0±0.0	1.0±0.0	1.0±0.0	1.0±0.0	1.0±0.0	1.0±0.0	1.0±0.0	1.0±0.0	1.0±0.0	1.0±0.0
5	1.1±0.1	1.6±0.3	1.2±0.1	1.2±0.1	1.0±0.1	1.3±0.2	1.6±0.2	1.1±0.1	1.2±0.2	1.1±0.1
10	1.7±0.1	2.1±0.4	1.2±0.1	1.2±0.1	1.0±0.1	1.8±0.1	2.1±0.4	1.1±0.1	1.2±0.2	1.1±0.1
15	2.0±0.5	2.9±0.3	1.2±0.1	1.2±0.1	1.0±0.1	2.1±0.4	2.9±0.3	1.1±0.1	1.2±0.2	1.1±0.1
20	2.7±0.7	3.3±0.3	1.2±0.1	1.2±0.1	1.0±0.1	2.8±0.6	3.2±0.4	1.1±0.1	1.2±0.2	1.1±0.1
25	3.3±0.3	3.5±0.3	1.2±0.1	1.2±0.1	1.0±0.1	3.2±0.4	3.5±0.4	1.1±0.1	1.2±0.2	1.1±0.1
30	3.5±0.5	3.7±0.3	1.2±0.1	1.2±0.1	1.0±0.1	3.4±0.6	3.7±0.4	1.1±0.1	1.2±0.2	1.1±0.1
60	4.2±0.1	4.5±0.2	1.2±0.1	1.2±0.1	1.1±0.1	4.1±0.1	4.6±0.4	1.1±0.1	1.2±0.2	1.1±0.1
150	4.8±0.2	5.3±0.3	1.2±0.1	1.2±0.1	1.1±0.1	4.8±0.2	5.6±0.2	1.1±0.1	1.2±0.2	1.1±0.1
