# Supplementary material for: Mode of action of the antimicrobial peptide Mel4 is independent of Staphylococcus aureus cell membrane permeability
Source: PLoS One. 2019 Jul 29;14(7):e0215703. doi: 10.1371/journal.pone.0215703 (PMC6663011; doi:10.1371/journal.pone.0215703)
Supplement: S7 Table — Bacterial lysis was determined as the decrease in OD620nm (in percentage) after treatment with 1X and 2X MIC of peptides. Data are presented as means (±SD) of three independent repeats performed in triplicate. (PDF) [file pone.0215703.s007.pdf]

S7 Table. Bacterial lysis of cells after addition of peptides. Bacterial lysis was determined as the decrease in OD_{620nm} (in percentage) after treatment with 1X and 2X MIC of peptides. Data are presented as means (±SD) of three independent repeats performed in triplicate.

Time (h)	*S. aureus* 31					*S. aureus* ATCC 6538				
	Melimine		Mel4		Buffer	Melimine		Mel4		Buffer
	1X	2X	1X	2X		1X	2X	1X	2X	
**0**	100±0	100±0	100±0	100±0	100±0	100±0.0	100±0.0	100±0.0	100±0.0	100±0
**2**	87±1	82±3	85±5	87±3	98±1	95±4	88±4	92±4	88±4	100±0
**6.5**	78±4	76 ±5	74±7	73±3	97±3	83±4	79±3	84±1	79±3	100±0
**24**	70±2	60±9	65±13	64±3	99±1	ND	ND	ND	ND	ND

ND=not determined
