# Supplementary material for: Mode of action of the antimicrobial peptide Mel4 is independent of Staphylococcus aureus cell membrane permeability
Source: PLoS One. 2019 Jul 29;14(7):e0215703. doi: 10.1371/journal.pone.0215703 (PMC6663011; doi:10.1371/journal.pone.0215703)
Supplement: S8 Table — The hydrolysis of peptidoglycan (PGN) was determined by measuring decrease in OD570nm. Data are presented as means (±SD) of three independent repeats performed in triplicate. (PDF) [file pone.0215703.s008.pdf]

S8 Table. Hydrolysis of peptidoglycan after addition of cell-free supernatants of cells that had been treated with peptides. The hydrolysis of peptidoglycan (PGN) was determined by measuring decrease in OD_{570nm}. Data are presented as means (±SD) of three independent repeats performed in triplicate.

Time (min)	*S. aureus* 31						
	Melimine+Bacteria	Mel4+Bacteria	Mel4+Buffer	Melimine+Buffer	Bacteria+Buffer	PGN+Buffer	Lysozyme (5mg/ml)
**0**	100±0	100±0	100±0	100±0	100±0	100±0	100±0
**30**	97±1	91±2	98±2	98±2	92±1	99±1	79±5
**60**	92±2	83±3	97±3	91±5	91±1	98±1	64±7
**90**	91±2	80±2	97±3	91±5	91±1	98±1	58±8
**120**	91±2	78±4	97±2	91±5	91±1	98±1	55±8
**150**	91±2	78±4	97±3	91±5	91±2	98±1	52±7
**180**	91±2	78±4	97±3	91±5	91±2	98±1	50±7

PGN=peptidoglycan
